# Editorial: The Functional Organization of the Auditory System

**DOI:** 10.3389/fnins.2016.00290

**Published:** 2016-07-07

**Authors:** Monica Munoz-Lopez, Yukiko Kikuchi

**Affiliations:** ^1^Human Neuroanatomy Laboratory, Medical Sciences, Medical School, University of Castilla-La ManchaAlbacete, Spain; ^2^Institute of Neuroscience, Newcastle University Medical SchoolNewcastle Upon Tyne, UK

**Keywords:** audition, anatomy, physiology, genetics, fMRI, plasticity, monkeys, rodents

Hearing, a central ability in our lives, allows us to distinguish the sound of birds from mosquitos, remember our favorite piece of music, have a conversation with a friend, and be alerted by a honk while crossing a road with our child. In other species, such as bats, this sensory modality is key for survival. Many researchers have dedicated their efforts to understand the auditory system. This research topic on the Functional Organization of the Auditory System brings some of the best scientific efforts to understand audition from genetic studies in rodents, electrophysiological recordings in rodents, bats, and monkeys as well as fMRI and translational research in humans. The following paragraphs aim to give a glimpse of the contents in this topic.

## Experimental work in rodents

Hearing loss among the aging or after injuries can be very debilitating. Fuentes-Santamaría et al. study experimentally induced conductive hearing loss in rodents and found changes in microglial cells, but not astrocytes, suggesting they may be the dynamic modulators of synaptic transmission in the cochlear nucleus immediately following unilateral hearing loss. Plasticity after injury of any component of the auditory system is of obvious clinical relevance. The thorough review presented by Gold and Bajo, reveals the potential of plasticity after injury, but also the sequels at different levels of the auditory hierarchy, from the brainstem to the cerebral cortex. Auditory driven alerting behavior is critical across species and has been further characterized by Gomez-Nieto et al. providing strong evidence that the cochlear root neurons-pontine reticular nucleus pathway mediates fast neurotransmission and binaural summation of the acoustic startle reflex in the rodent. Auditory hallucinations are a distinct symptom of schizophrenia. Nakao and Nakazawa, present a rodent model of this disorder and show that the “paradoxically” high spontaneous LFP activity of the primary auditory cortex in the absence of external stimuli may possibly contribute to the emergence of schizophrenia-related aberrant auditory perception. These authors suggest that NMDAR hypofunction in cortical GABAergic interneurons leads to two temporally distinct, brain state-dependent LFP deficits in the A1 cortex. Sotoca et al. present a surprising result in a transgenic P23H-1 rat model that opens up new strategies of study on deaf-blindness implicating a direct effect on rhodopsin mutation.

## Electrophysiology, anatomy, and fMRI in monkeys

Closer to humans, non-human primates like rhesus (*Macaca mulatta*) or cynomolgus (*Macaca fascicularis*) monkeys have a well-defined temporal lobe with superior temporal plane, where the primary auditory cortex lies. This proximity of the monkey brain to humans offers an exceptional opportunity for better understanding the human nervous system, and the auditory, in particular, yielding some unique access to information on the evolution of audition toward speech. A research on the electrophysiological properties of neuronal responses within the primary and belt areas of the auditory cortex to simple and complex auditory stimuli (Kikuchi et al.) has shown that harmonic features, such as relationships between specific frequency intervals of communication calls, are processed at relatively early stages of the auditory cortical pathway, but preferentially in LB. Detailed anatomical data on the feed-forward and feed-back arrangement of circuits of primary, belt, and parabelt areas presented by Hackett et al. showed the convergence of feedforward inputs into rostral medial belt from middle lateral belt (ML) and caudal parabelt (CPB). This was surprising given that CPB is at a higher stage of the processing hierarchy, with mainly feedback projections to all other belt areas. These data have stimulated a revision of the conventional model. Joly et al. addresses methodological issues of fMRI and anatomical mapping by means of combining information from cortical folding, micro-anatomy, surface-based atlas and tonotopic mapping. The fMRI study by Ortiz-Ríos et al. identifies the network for species-specific vocalization processing in the auditory ventral pathway, and they highlight the involvement of the anterolateral belt and parabelt areas. This fits well with our own anatomical data showing that the rostral superior temporal gyrus sends convergent input to the dorsal half of the temporal pole (Muñoz-López et al.). It is particularly in this region of the dorsal temporal pole that neurons show working memory-related responses (Bigelow et al.). Furthermore, even if monkeys can hold auditory stimuli in working memory, their ability to keep them for longer delays is very limited. The anatomical work by one of the editors (Muñoz-López et al.) aims to explain this poor auditory long-term memory ability by demonstrating that the connections from the dorsal temporal pole to medial temporal cortex bypass most of area 36 of the perirhinal cortex, to directly reach area 35, entorhinal, and posterior parahippocampal cortices. This contrasts with the highly dense projections from the visual cortex to area 36 of the perirhinal cortex, and may explain, at least in part, the poorer auditory memory in monkeys compared with vision. These anatomical data support the hypothesis that auditory stimuli can access the medial temporal cortex if intermingled with information from multimodal areas and this may guarantee the persistence of memory traces that include auditory information (Muñoz-Lopez et al.).

Outside the temporal lobe, several prefrontal areas have been put forward as important for auditory processing. The review by Medalla and Barbas points to area 10 as the one receiving the densest auditory input in monkeys. On the other hand, Plakke and Romanski present a complementary view whereby different areas of the dorsolateral and ventrolateral prefrontal cortex participate in different cognitive tasks depending on task demands. Data from electrophysiological recordings by Bigelow et al. highlight the possibility that match-enhancement of neural responses observed in A1 and dorsal temporal pole during working memory may reflect top–down feedback from prefrontal cortex.

From the prospective of motor-auditory interactions, Lovell et al. show that the red nucleus appears to receive inputs from a very early stage of the ascending auditory system, suggesting that sounds might influence the motor control exerted by this brain nucleus.

The study by Lui et al. in marmosets show that, although A1 neurons are sensitive to interaural level differences (ILDs), the ILD sensitivity depends on stimulus types (species-specific calls vs. pure tones). They also show that ILD sensitivity was heavily dependent on binaural levels, suggesting that with the lack of binaural level invariant encoding in A1 neurons, A1 neurons seem to encode auditory space using population codes.

## Audition in bats

Washington and Tillinghast show the findings in bats to support that the hemispheric specialization for speech and music in humans is based on hemispheric specialization for temporal and spectral resolution.

## Auditory research in humans

Patient studies are of critical importance when studying the function of the nervous system. The patient with a tumor resection in the ventral precentral gyrus presented by Tanji et al. demonstrates that a lesion restricted to this auditory-responsive area is enough to cause apraxia of speech. The relevance of this result roots in that this lesion is sufficient to impair the execution of fluent speech, but leaves speech perception/comprehension intact. This result has important theoretical implications for the motor Sensory vs. Motor theories of speech. This study takes parts and bets for the sensory theory of speech. Lahav and Skoe present their work in neonatal intensive care units with the aim of better understanding the impact of the noisy sound environment on the developing auditory system as an important first step in meeting the developmental demands of preterm newborns. Su et al. present a novel method to resolve spatial organizations of human auditory cortex using a combination of EEG, MEG, and fMRI data and multivariate pattern analysis.

The importance of individual differences in human primary auditory cortical maps is addressed by Moerel et al. by examining tonotopic maps at a single-subject level to detail the topography of primary and non-primary areas. They merge multiple maps indicative of anatomical (i.e., myelination) as well as of functional properties (e.g., broadness of frequency tuning) to better identify auditory cortical areas in individual human brains. Individual differences add up with dynamics of auditory representations in humans in the fMRI data on attentive vs. inattentive auditory events. Amaral and Langers show the suppressive binaural interaction during a dichotic listening, which illustrates the dynamic properties of our auditory processing.

Of clinical relevance is the study by Johannesen et al. Identifying the multiple contributors to the audiometric loss of a hearing impaired listener at a particular frequency is becoming gradually more useful as new treatments are developed.

This research topic reunites a wealth of studies of diverse animal species including humans and it is our hope that they contribute to better understand mechanisms underlying audition and to achieve real-life applications in clinical populations.

## Author contributions

MM was invited to prepare this RT and invited Dr. YK to be co-editor in it. We both prepared and discussed a list of guests-authors, invited them, revised their manuscripts and handled their revisions.

## Funding

This study was supported by NIMH/IRP grant BFI 2003-09581 and the Spanish Ministry of Science and Innovation grant BFU 2006-12964.

### Conflict of interest statement

The authors declare that the research was conducted in the absence of any commercial or financial relationships that could be construed as a potential conflict of interest.

